# Selections

**Published:** 1892-06

**Authors:** 


### *SELECTIONS.*

---

Brain Surgery from the Physician's Point of View.—In an exhaustive article on this subject in the *Sammlung Klinischer Vorträge* Dr. Hermann Sahli thus concludes:

(1.) Horsley recommends deligation of the carotid in every case of spontaneous cerebral hemorrhage to which the practitioner has been summoned within the first four hours. Such treatment would be contrary to medical opinion, and, in the majority of cases, would either be useless or do harm.

(2.) The author would, in the interest of patients, protest most strongly against the practice of trephining in all cases of headache which may have resisted other methods of treatment. It would be rash, without exact diagnosis of a local cause of the headache, to submit the patient haphazard, and without the smallest guarantee for success, to an operation which, in spite of all antiseptic precautions, is likely to be attended by risk.

(3.) Exploratory trephining should not be performed unless it be comparatively necessary, as this operation is not free from danger.

(4.) Tumors of the brain can be extirpated only in a diminishing minority of cases.

(5.) Horsley recommends that in suitable cases a cerebral tumor be extirpated as early as possible, and that when the diagnosis has been established, the operation be not delayed beyond an interval of six weeks, if no alleviation has been attained by other means. This postulate may be theoretically correct, but, practically, an opposite view might be taken if attention be paid to the very variable condition of the patient's health in cases of brain tumor, and to the undeniable danger of the operation.

(6.) The proposal to deal with gummatous tumors of the brain is based on erroneous conclusions as to the curative results of a long-maintained antisyphilitic treatment. In the present position of cerebral surgery the subject of a gumma of the brain has a much better chance with medicinal than with operative treatment.

(7.) The prospects of surgery are more favorable with regard to abscess than to tumor of the brain. In the treatment of the former much good has already been done by operation, and with still more precise methods of local diagnosis further progress may be made.

(8.) Of the different forms of epilepsy, it is only, as a rule, in well-marked cortical (Jackson's) epilepsy that surgical treatment is likely to be attended with good results. Still, even in cases of this variety, the results of operation have not been constant. With improvements in technical details (improved facilities for making out the affected centre by electric currents and more precise localization of the primarily convulsed region by minute clinical observation) still better results may in all probability be attained.

(9.) Operations for relieving the brain of abnormal pressure in incurable affections of this organ claim more attention than they have hitherto received, since the purely symptomatic indications thus fulfilled are of very great consequence. By such treatment adapted to symptoms, the headache associated with cerebral tumor and with tuberculous meningitis may be relieved. Trephining as has been performed wiith such an object in view, with division of the dura mater for the production of cerebral prolapse, with or without removal of the protruded portion of brain, is attended with certain risks, and is probably less efficacious than puncture of the ventricles. The trials that have hitherto been made of this plan are too few in number to permit any correct judgment being formed as to the utility and the dangers of puncture of the ventricles. Puncture made without any free division of the dura mater, and by penetrating the bone and membranes with an instrument of sufficient size to allow room for the subsequent introduction of a small trocar, seems to have certain advantages over puncture of a portion of brain surface freely exposed at the bottom of a large opening made in the cranial wall.

(10.) In opening the skull the trephine crown should always be used, and never the chisel, as by the concussion set up by the application of the latter instrument the danger of operative violence is probably increased to an unnecessary degree.

(11.) The surgical treatment of mental disorders, in the present state of our knowledge, is wanting in a needful scientific basis.—*British Medical Journal.*

---

ACUTE IDIOPATHIC INFLAMMATION OF THE GLANDS OF THE NECK.—Neumann (*Berliner klinische Wochenschrift*), in a paper read before the Berlin Medical Society, calls attention to the fact that in the books upon children's diseases the inflammation of the glands of the neck is looked upon as being an infectious disease following diphtheria and scarlet fever. He says that Pleiffer described a disease which he called glandular fever. The author has observed an acute idiopathic inflammation of the deep glands of the neck at the angle of the jaw as constituting a well-characterized clinical picture. The disease occurs in childhood and especially during the first years of life. Of twenty-seven cases observed, eleven were one year old or under. The youngest was ten weeks old, ten were in the second year, and, of the other six, two were in the fourth year. Nineteen were boys and eight were girls. The younger children were mostly well developed; of the older some were affected with rachitis or slight scrofulous adenitis. The typical cases were suddenly seized while in full health, or they perhaps had a slight cold in the head, or in a few cases a slight cough previous to the onset of the disease. The fever began quickly, with considerable intensity, as shown by heat, increased thirst, general restlessness and disturbed sleep, sometimes in small children vomiting, and in the older children severe headache. In three cases the rectal temperature was 103°. In the same or the following day, only exceptionally later, glandular swelling was noticed in the neck, which quickly became of huge dimensions. Usually the fever was moderately high, varying between 101½° to 103°. Pulse and respiration were correspondingly increased, but held the normal relation to each other. Examination of the chest and abdomen was negative. In typical cases the throat is not at all or only slightly reddened, while the outside of the neck, filling the space between the lawer jaw and the clavicle, on one side, is a regular, reddened, painful swelling. It begins at the junction of the head with the neck; and in severe cases, in the course of the phlegmonous process, the deep upper cervical glands are united in one mass, sometimes reaching the size of an orange. The mass sometimes measured, from the anterior to the posterior part, two to three inches. In a few cases the corresponding glands on the opposite side of the neck were enlarged, but to a less extent. The head was fixed and sometimes bent toward the side of the swelling. The right side of the neck was more frequently affected than the left. The fever disappeared sometimes very suddenly, as early as the second day. After the fever fell, the swelling was less painful and began to disappear. Resolution took place thirteen times, generally being complete in two to four weeks. In thirteen of the cases the glands suppurated, the time of suppuration varying from the third to the twenty-second day. The abscess was fully healed, five to seven weeks from the beginning of the disease. In one case only did a chronic lymphadenitis persist. In the beginning of the disease ice-packs were placed upon the swelling, and later the swelling rubbed with an iodide of potassium ointment with green soap in the proportion of eight to four. When suppuration took place a poultice was applied.—*Univ. Med. Mag.*

---

TREATMENT OF GRANULAR LIDS.—Dr. F. Buller, Professor of Ophthalmology at McGill University, in the Montreal *Medical Journal*, says:

Two remedies which I have had most satisfaction from in the last two years are jequirity and the operation of squeezing out the granulations. The former plan of treatment is only applicable in a certain class of cases, such as were formerly deemed suitable for treatment by inoculation. That is, old, inveterate cases with considerable corneal vascularity and rather firm trachomatous infiltrations, the conjunctiva being rather the reverse of moist and succulent.

The large and numerous round, or flattened and smooth, fibrous granulations are not amenable to the jequirity treatment. In favorable cases, one or two applications of a four per cent. solution of jequirity freshly prepared sets up a pretty active croupous conjunctivitis, which often becomes distinctly purulent in character, and the subsidence of this in a few days is followed by a rapid and often complete recovery. We must, however, be prepared to meet with disappointment in some instances, the granulations and pannus failing to disappear where the indications seem clear for the use of this drug, and the reaction it caused quite after the normal type. I have only twice seen it cause troublesome ulceration of the cornea, and occasionally a dacryocystitis.

The operation of pressing out granulations is best done with Prince's ring forceps. The larger and more pulpy and gelatinous-looking the granulations are, the more thoroughly they can be eradicated in this way. Immediately after the operation the conjunctiva should be freely washed with a pretty strong solution of perchloride of mercury, and a weaker solution used several times daily until the abraded conjunctivitis heals. The results of this treatment are, as a rule, exceedingly satisfactory.

I do not mean to imply that the old standard remedies for granular ophthalmia are to be supplanted by the more recent ones, for they still play an important part in the treatment of more recent cases, and, in fact, the judicious use of silver, copper sulphate, alum, oxide of mercury, tannic acid, and some other remedies will generally suffice to cure ordinary recent cases, and it is only when too zealously and persistently employed that they are likely to prove hurtful. This is, perhaps, especially true of the silver and copper preparations, which should never be persevered with in the face of an augmenting irritation.

The true secret of success in the treatment of this disease in any of its forms lies in a careful observation of details, both local and general, and a nice adaptation of the requirements of each case, rather than any attempt at routine practice.—*Medical Age.*

PLEA FOR EXPLORATIVE INCISION IN ASCITES IN WOMEN.—Of these causes of ascites but three concern us here: The existence of neoplasms within the peritoneal cavity, chronic peritonitis, and that diseased condition of the peritoneum which has been styled, "tubercular peritonitis." I allude to the last pathological condition thus doubtfully because it behaves in a manner so unlike tubercular disease developing in other parts of the body under the influence of surgical interference.

These three causes of ascites will, as to authenticity, be disputed by no one. All practitioners have in *post mortem* examinations met with instances of the second and third causes. As to the first, we see it in rare cases active with all varieties of solid tumors of the uterus and of fluid ones of the ovary. This is so well recognized as a fact that it requires no further consideration at my hands, so far as the general proposition is concerned. The special proposition which I would make in reference to them is this: that some cases of excessive ascites which by repeated tappings prove fatal are due either to chronic or tubercular peritonitis, which is recovered from by opening the peritoneal cavity and draining it thoroughly, or to the existence of insignificant uterine or ovarian tumors which are two small for recognition, unless specially and carefully sought for, and the removal of which relieves the fluid accumulation, which by its exhausting influence destroys life.

I have met with a number of cases in which I have succeeded in completely curing aggravated cases of ascites after tapping had been repeatedly resorted to, and after all hope of recovery had been given up.

Explorative incision, practiced with antiseptic precautions now at our disposal, is not a dangerous procedure. If a good result attend it, a saving of life is the outcome; if it reveal an incurable or organic disease, no evil will usually accrue; and even if a fatal issue should be its consequence, we will be forestalling death by a short time only, in a praiseworthy effort at the securing of life.

It appears to me that with the evidence which is before us we should accept the following as a rule for practice: *In every case*

*of ascites in woman, the propriety of explorative abdominal incision should always be carefully considered; not with the view of establishing a certain diagnosis alone, but with the reasonable hope of effecting in exceptional cases a cure.—T. Gaillard Thomas, M. D., N. Y. Jour. of Gyn. and Obstet.*

---

AN OBSIETRICAL BUNDLE.—This bundle I have found very useful. I have such a bundle prepared for every obstetric case, and its cost, seventy-five cents, is more than made up by the saving of time and subsequent visits. It contains the following:

1. One square yard of rubber cloth to be placed under the patient's hips and thighs—rubber side up of course.

2. One square yard of cotton flannel to be placed on top of the rubber, between it and the patient's body. In this way I make sure of having the bed protected and kept clean, and an aseptic environment, and the rubber can be quickly arranged to carry off the fluids into a suitable receptacle in case of operative procedures.

3. A number of pieces of cheese cloth to be used as small towels, and also, when dampened with bichloride solution, as pads for the vulva.

4. A new and clean nail brush for each case. The brushes cost three cents, and hence one can afford a new one each time.

5. Safety pins.

6. A narrow bobbin, consisting of three strands, for ligating the umbilical cord.

7. An obstetrical eye bandage. This consists of a strip of cheese cloth, the two edges of which are rolled in and then doubled over a second time. While waiting for the pulsation of the cord to cease I wipe out the baby's eyes, and wrap this bandage around the head and eyes, and pin it. When this is not done the child often rubs its dirty fingers into the eyes before the attendants have had time to wash the child. Since I have adopted this plan I have never had any cases of ophthalmia neonatorum.

8. A small wooden vial containing tablets of bichloride of mercury. I prefer these small ones to the larger size, as they are just sufficient for each dressing without splitting the tablet.—*George E. Abbott, M. D., in Post Graduate.*

CONDITIONS UNDER WHICH CURE IN CONSUMPTION IS POSSIBLE.—In a recent address before the Medical Society of London, Dr. J. Burney Yeo emphasizes *the conditions under which a cure in consumption is possible*, and protests against testing our new remedies in cases in which the conditions for cure no longer exist. The following facts are emphasized: The importance of an early recognition of the disease before the tuberculosis has developed into phthisis. This state may be recognized to the extent of probability, not to the extent of certainty. Certainty is reached only when the bacillus can be detected in the expectoration, and the bacillus cannot be detected until there is destructive ulceration of the lung and small cavity formed communicating with a bronchial tube; in short, not until the phthisical process is somewhat advanced. The early occurrence of hæmoptysis is regarded as favorable, inasmuch as it impressively calls attention to this early stage. Those cases are also regarded as more hopeful in which there is a natural tendency in the evolution of the tubercle to fibrous change, or in which there is an absence of tissue-sensitiveness, or irritability, evidenced by the tendency to acute inflammatory reaction to the bacillary infection. Another favoring condition is the absence of marked hereditary predisposition, and the possossion of a sound, vigorous constitution before the accidental infection. Lastly, we must have a sustained functional activity of the organs of digestion and assimilation. The phthisical patient who cannot digest and assimilate nourishment is in great peril.

Among the therapeutic conditions necessary or favorable to the cure of phthisis are: 1. The administration of food in suitable quantity, and in such forms and methods as shall insure its digestion and assimilation. 2. The possibility of a life much in the open air, either in the country or on the sea, where there is much sunshine, and where the air is dry and pure. 3. A continuous and strict attention to minute details of daily life and hygiene. A therapeutic agent, universally esteemed in promoting curative changes in tuberculous deposits in the early stage, is counter-irritation, repeated and continuous. Furthermore, he says of antiseptics given internally that he has seen none so uniformily beneficial as creasote or guaiacol. It has held its ground better than any other antiseptic agent that has been applied to the treatment of phthisis, and has steadily gained in favor.—*International Med. Magazine.*

---

THE ETIOLOGICAL RELATION OF SYPHILIS TO TABES.—Erb, in a recent article in *Berliner klinische Wochenschrift*, Nos. 29 and 30, 1891, gives a critical analysis of some 370 cases of tabes observed by him since his last paper on the subject in 1883. He finds his former conclusions fully supported by the new list. Of these cases, 300 were men in the higher walks of life, and of them 89 per cent. had been previously infected, 63.3 per cent. having had positive secondary symptoms and 25.7 per cent. had chancre without observable secondary symptoms. Of the remaining 11 per cent., 8 per cent. had had gonorrhœa, most of them several times. They had therefore been exposed to infection by syphilis. It may then be safely said that but 3 per cent. were free from the suspicion of syphilis. Of 50 cases of men from the lower walks of life, 70 per cent. had been previously infected, and of these 52 per cent. had secondary symptoms, and 24 per cent. had chancre only. Of the 24 per cent. alleged to be without previous infection, ignorance may well have made their statements in many instances untrustworthy, and besides several had had gonorrhœa and of these latter one had a cicatrix on the penis with enlarged glands; the wife of one had an abortion and three still-births, etc. Hence, the number of the infected ones should probably be greater. Of women, his list has 19 cases, 10 being from the lower and 9 from the higher ranks of life. Of these cases 47.4 per cent. were positively syphilitic and 42.1 per cent. were syphilitic almost without doubt. Of those without suspicion of syphilis there were 10.5 per cent. Of all the cases in which any information could be obtained concerning the apparent cause of the disease, there were only 11 per cent. in which syphilis had not been present and half of these justified a suspicion. The influence of exposure to cold, of excessive fatigue, of sexual excess, of trauma and of neuropathic heredity or of combination of these, without syphilis, must be very slight, although Erb will not agree with Strumpell, Binswanger and others to hold it as of no value, but the table in which these causes as given in the history of the cases appear in syphilitic individuals is startling in its percentage proportions of such cases. He concludes that syphilis is by far the chief and most important cause of tabes, that other injurious influences play only a very subordinate rôle, but that in not a few cases they arouse it into this specialized activity.—*Exchange.*

---

TYPHOID FEVER.—Dr. E. L. B. Godfrey, of the Cooper Hospital, Camden, N. J., lays much stress upon the following points in the treatment of typhoid fever:

1. Absolute recumbency in order to preserve the strength of the patient.
2. The early use of the bed-pan for rectal dejections.
3. Liquid food from the beginning, and continued for one week after cessation of fever.
4. Great care to avoid overfeeding during the first week, and the use of peptonized milk during the course of the fever.
5. Feed the subject regularly and at night.
6. The use of internal disinfectants, particularly the sulpho-carbolate of zinc or sub-iodide of bismuth in five-grain doses every three hours, in order to allay fermentation and to disinfect the stools.
7. Turpentine in the latter stages, when tongue is dry and tympanites marked.
8. Strychnia for heart tonic, with digitalis.
9. Morphia for headache during the first week.
10. Stimulants regularly administered when pulse reaches 118, or when tympanites, subsultus tendinum or cold extremities are present.
11. Frequent sponging to allay high temperature.
12. Make a special point to combat rapid pulse, high fever, tympanites, subsultus tendinum, and cold extremities.

And the editor of the *Medical World* adds:

13. Antiseptic colon flushing, to prevent the absorption of septic material.

OPERATIVE INDICATIONS FOR STONE IN THE BLADDER.—*Median Perineal Lithotomy.*—Adults; very small stone; irritable and contracted bladder; marked cystitis; moderate-sized prostate.

*Lateral Perineal Lithotomy.*—Children under five years of age, medium-sized stone; adults where there is present a very bad cystitis, contracted and irritable bladder, medium-sized stone. Under these circumstances the lateral is preferable to the median operation. Owing to the wound being larger, the bladder can be better drained and the stone more easily extracted.

*Suprapubic Lithotomy.*—Adults and children; very large and hard stone; stone, the nucleus of which is foreign body; marked enlargement of the prostate. Where the latter is present this operation gives the surgeon, after having removed the calculus, the opportunity of doing a partial prostatectomy, which operation enables the patient to live without the use of the catheter, or least renders the passage of the instrument less difficult.

*Litholapaxy.*—Absence of Bright's disease; absence of cystitis to any degree; a non-contracted and retentive bladder, one which, if in an adult, will contain and retain six to ten ounces of boracic acid solution, or the equivalent amount of urine, and, if a child, from four to six ounces; a non-irritable urethra. This is determined by passing a steel bougie, the size of the urethra, for two or three days before the operation.

Should the urethra be the seat of a stricture or strictues, there being no other contraindication to crushing, they should be either cut or dilated, depending upon their location; a prostate not too large to embarrass the ready passage of a large lithotrite and the evacuating catheter; a urethra allowing the ready passage of the lithotrite and evacuating tube; a stone neither too large nor too hard.—*Dr. John B. Deaver, International Med. Magazine.*

---

TREATMENT OF DYSENTERY.—Since dysentery is undoubtedly a local and specific disease, by far the most rational treatment is by irrigation of the large intestines in severer forms. Many cases will recover almost immediately after an irrigation with cold or ice-water, if the lower bowel be thoroughly irrigated and all of its contents removed. Wood highly recommends the treatment of specific dysentery by injection of nitrate of silver, 1 drachm to pint of water three times daily, and claims some surprising cures. A very successful way of irrigating is by injecting as much water as possible with a dram of alum to the pint. Salicylic acid is in this way often a benefit, but carbolic acid cannot be used on accounnt of its toxic effects. Bichloride of mercury has also been frequently used as well as all the other antiseptics. If there is a specific in the treatment of dysentery it is pulverized ipecac. In all acute cases give from 30 to 60 grains every four hours, as it must be given in decided doses to obtain its effects. My method of using it is to give one dram, and if necessary repeat in six hours. It causes a great deal of nausea, and sometimes vomiting for two hours. Then the patient breaks out in a profuse perspiration, the pulse becomes fuller, softer and more regular, and tenesmus and abdominal pains cease and there are no more stools for from eight to twenty-four hours. Ipecac has all the advantages of mercurial purgatives without their irritating action ; all the results of sudorifics without their uncertainty ; all the benefits of opium without any of its disadvantages. Should the remedy fail to be of value in forty-eight hours, it should be discontinued and irrigations used. Turpentine, internally and externally, has had its advocates ; also astringents, such as tannic acid, kino, catechu, krameria, acetate of lead, and nitrate of silver ; also boric acid, opium and its preparations, and quinine. All other things failing as a cure in chronic cases, a permanent change of climate should be advised.—*Am. Pract. and News.*

---

TREATMENT OF GONORRHŒAL VAGINITIS.—The woman, if possible, should be kept in bed, and salol and an alkali administered internally for the relief of painful micturition. A lotion of bichloride solution 1 : 5,000 is used for bathing the outer parts and for injecting into the vagina three or four times a day for the first three days. After this the strength is reduced to 1 : 10,000. If this injection be painful carbolic acid solution is substituted for it. The injections should be taken with the patient in a recumbent posture. About the third day a speculum can be used and the cervix exposed to view. The parts may then be swabbed with strong carbolic acid or with a solution of bichloride of mercury, and this followed by dusting the parts with powdered iodoform. Later on a five-per-cent. solution of nitrate of silver may be painted over the cervix and vagina, and the vagina tamponed with iodoform gauze so as to separate its walls, the ends of the tampon being brought down to the vulva. If these are changed every two days the discharge will usually cease within a week or ten days. Before discharging the case as cured, never omit to examine the glands of Bartholini, and if any pus can be made to exude from them you may rest assured that the disease has not been eradicated. They should then be incised under cocaine anesthesia and touched with strong carbolic acid. If there be purulent discharge from the cervix and the Nabothian glands are distended, curette them and apply pure carbolic acid, using the iodoform tampon to prevent reinfection of the vagina. —*Am. Journal Obstetrics.*

---

CATARRHAL ENTERITIS.—In the *British Medical Journal* (April 16) Dr. Bottentint describes the treatment of this disease, as it is carried out by him at Plombieres. The disease has received several names, as glutinous diarrhœa, tubular diarrhœa, glairy enteritis etc. The author prefers the above. The treatment consists, first of tepid baths. These exercise a calming and sedative action on the nervous phenomena, which many of these patients exhibit, and do great good. But the more important agent is the continual irrigation of the colon by mineral water, or, as it is called, the *douche ascendante.* These can be given to patients without subjecting them to an uncomfortable position or exposing them to fatigue. Fountain syringe should be used and too great pressure avoided. The prolonged contact of the mineral water with intestine is an internal bath which produces excellent effects on the inflamed membrane. The pains decrease. The mucous secretions are modified and expelled much sooner and with greater ease than they otherwise would be.

